# Supplementary material for: Tumor-Colonizing E. coli Expressing Both Collagenase and Hyaluronidase Enhances Therapeutic Efficacy of Gemcitabine in Pancreatic Cancer Models
Source: Biomolecules. 2024 Nov 17;14(11):1458. doi: 10.3390/biom14111458 (PMC11591662; doi:10.3390/biom14111458)
Supplement: Supplementary file 1 [file biomolecules-14-01458-s001.zip › biomolecules-3310913-supplementary-re.pdf]

A

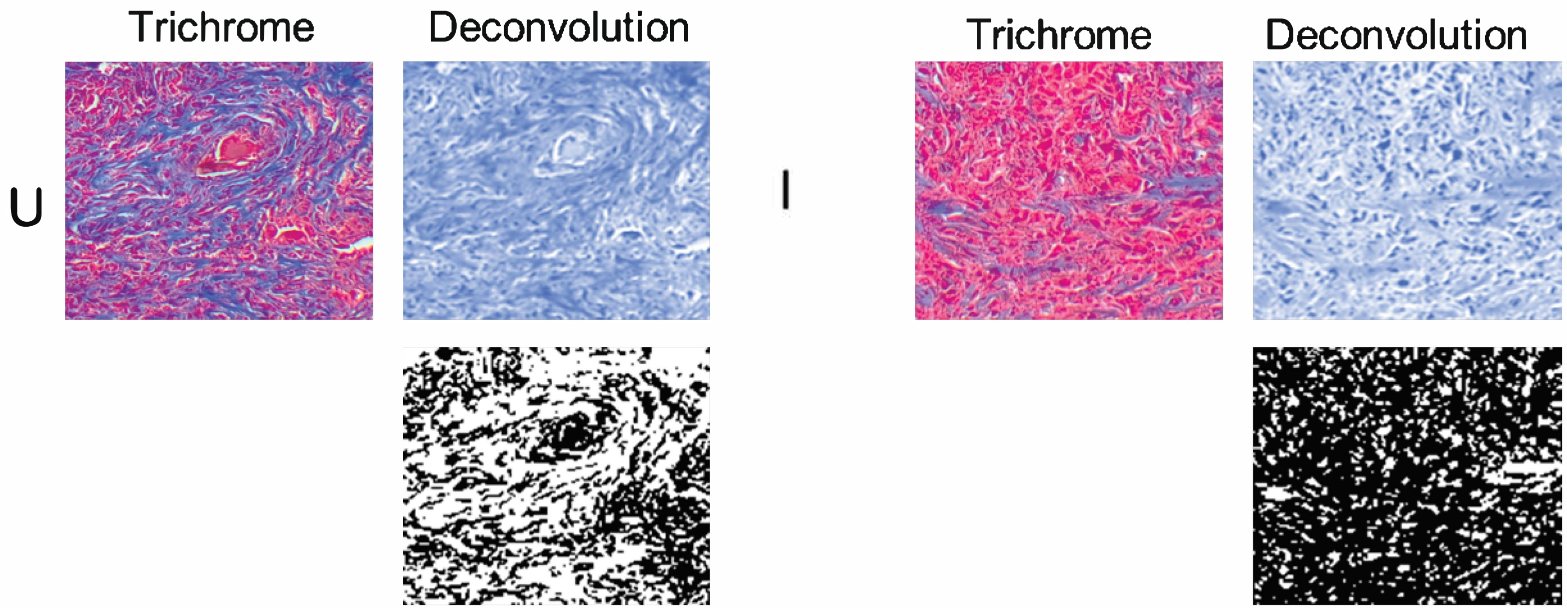

B

	Mean	IntDen	RawIntDen
U	93.771	1.10E+06	1.10E+06
I	89.932	3.81E+05	3.81E+05

Supplemental Figure S1. Representative analysis showing deconvolution of trichrome staining for collagen density within tumor sections. Serial sections of KPC tumor treated overnight with BL21-TAN under uninduced or induced conditions at 37°C. Sections were then stained by trichrome and deconvolution of collagen (blue/white) was performed **(A)** and collagen density was measured **(B)** using ImageJ.

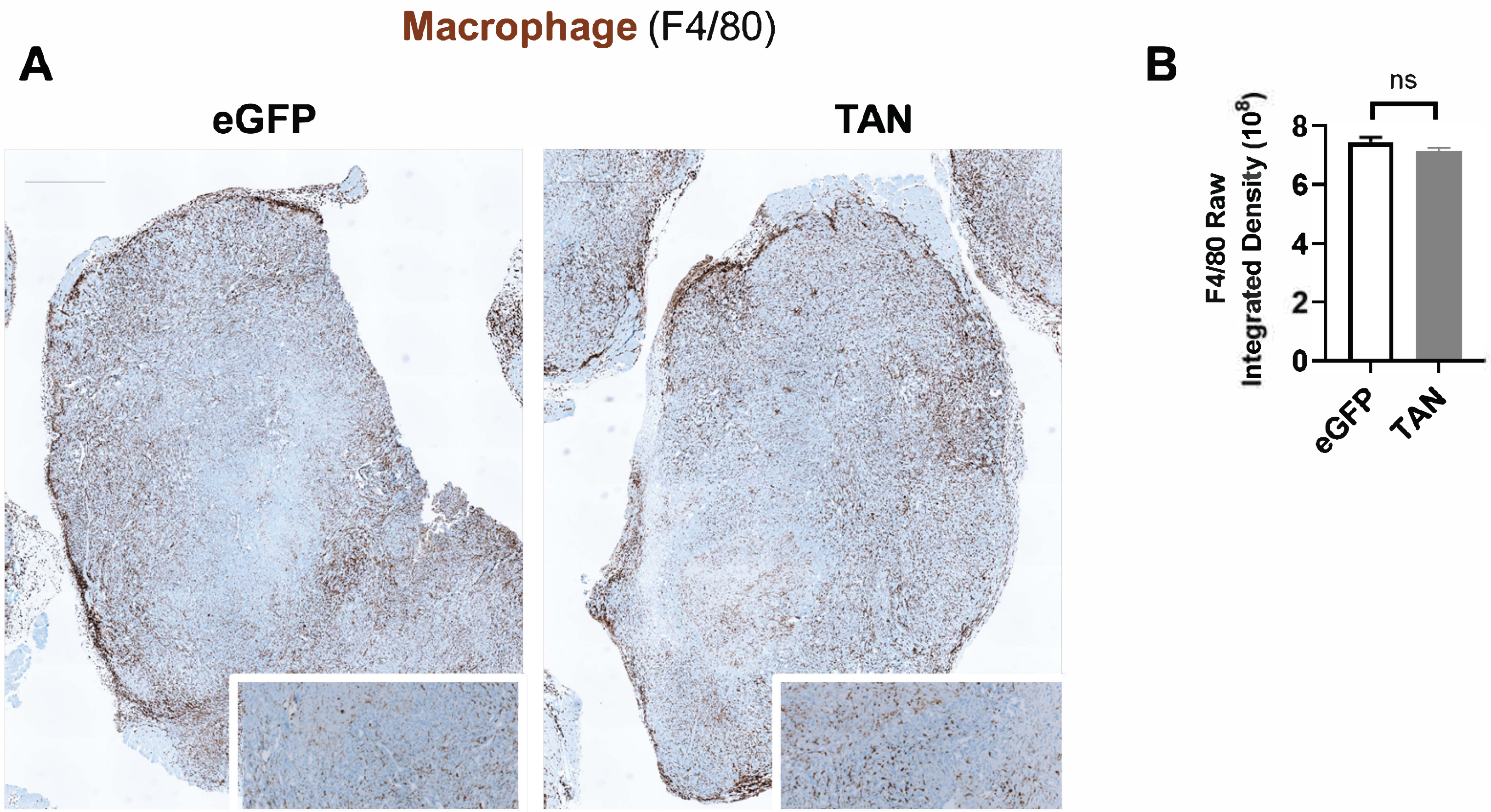

Supplemental Figure S2. In vivo macrophage infiltration in BxPC3 tumors following BL21-eGFP or BL21-TAN treatment. NSG mice with subcutaneous BxPC3 xenografts (6-8mm diameter) were injected with $5x10^7$ colony-forming units (CFUs) of BL21-eGFP control or BL21-TAN for three consecutive days by intravenous route. Twenty-four hours following the final injection, mice given BL21-TAN were administered 40 mg L-arabinose intraperitoneally. Tumors (*n*=8) were collected 48h post-induction and serial sections were evaluated for macrophage infiltration by staining for F4/80 **(A)**. Regions (box) from each image were magnified for greater resolution of collagen and HA (inset). Random fields (*n*=15, 10X objective) from each treatment group were used for deconvolution analysis to quantify macrophage infiltration in multiple tumors **(B)**. Data are expressed as mean ± error of mean. *ns = no significance*, *t*-test. Scale bars = 1 mm.
