# Supplementary figures and images for: Tumor-Colonizing E. coli Expressing Both Collagenase and Hyaluronidase Enhances Therapeutic Efficacy of Gemcitabine in Pancreatic Cancer Models
Source: Biomolecules. 2024 Nov 17;14(11):1458. doi: 10.3390/biom14111458 (PMC11591662; doi:10.3390/biom14111458)

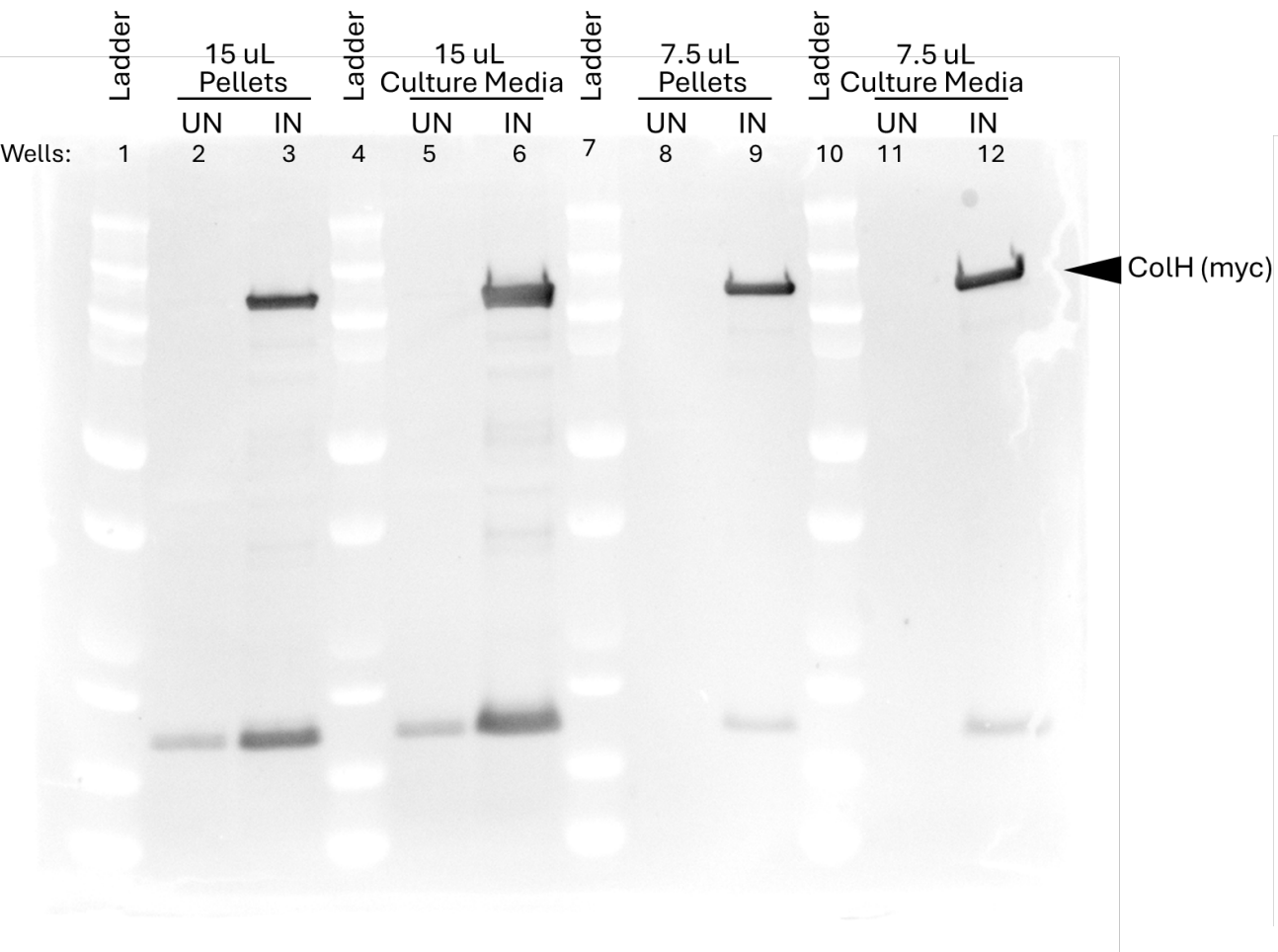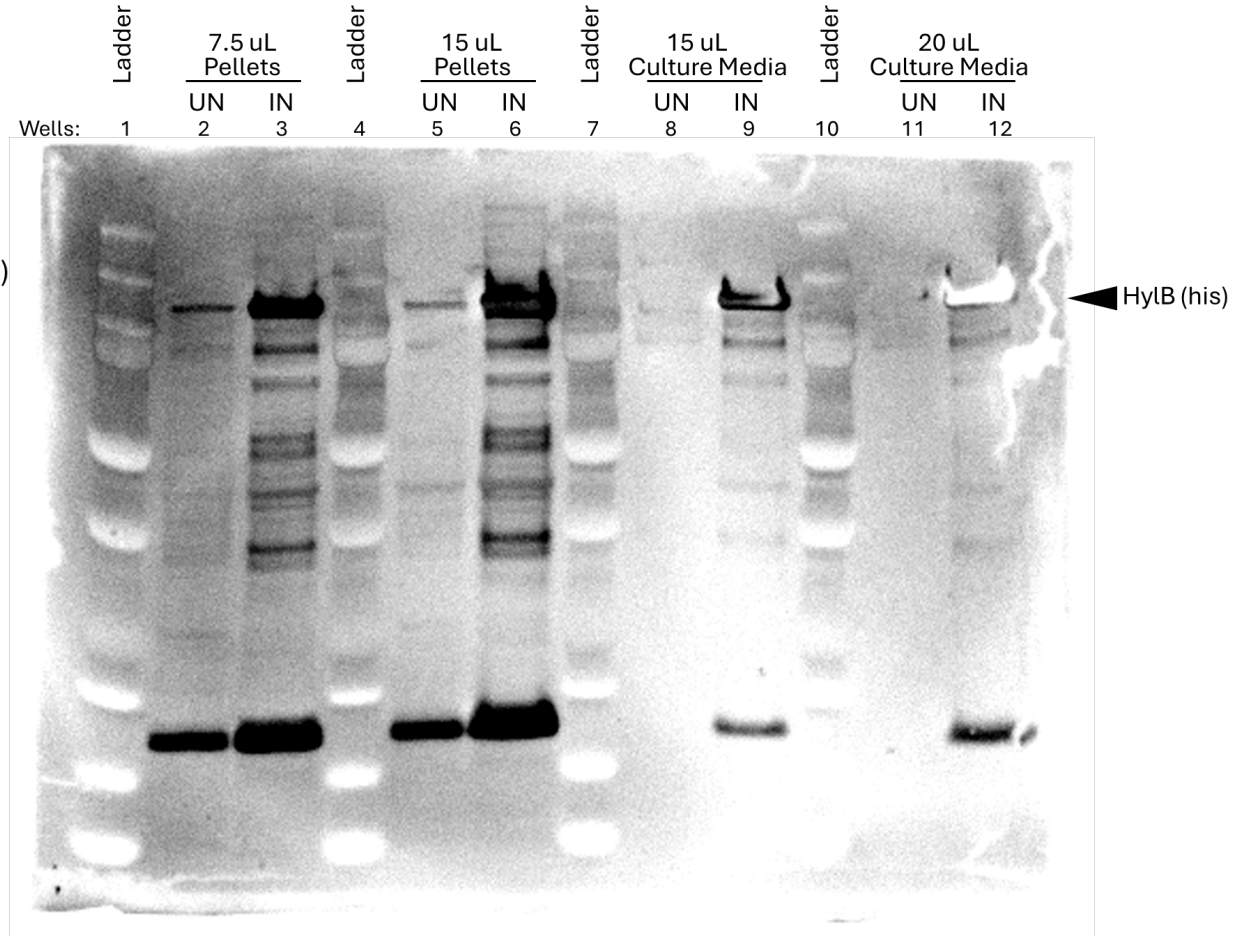

Supplement: Supplementary file 1 [file biomolecules-14-01458-s001.zip › biomolecules-3310913-original images.pdf]
